# A deep convolutional neural network to predict the curve progression of adolescent idiopathic scoliosis: a pilot study

**DOI:** 10.1186/s12891-022-05565-6

**Published:** 2022-06-24

**Authors:** Yasuhito Yahara, Manami Tamura, Shoji Seki, Yohan Kondo, Hiroto Makino, Kenta Watanabe, Katsuhiko Kamei, Hayato Futakawa, Yoshiharu Kawaguchi

**Affiliations:** 1 Department of Orthopaedic Surgery, Faculty of Medicine, University of Toyama, 2630 Sugitani, Toyama 930-0194 Japan; 2 Department of Molecular and Medical Pharmacology, Faculty of Medicine, University of Toyama, Toyama, Japan; 3 Department of Radiological Technology, Graduate School of Health Sciences, Niigata University, 2-746 Asahimachi-dori, Chuo-ku, Niigata, 951-8518 Japan

**Keywords:** Scoliosis, Risk prediction, Machine-learning, Deep convolutional neural network

## Abstract

**Background:**

Adolescent idiopathic scoliosis (AIS) is a three-dimensional spinal deformity that predominantly occurs in girls. While skeletal growth and maturation influence the development of AIS, accurate prediction of curve progression remains difficult because the prognosis for deformity differs among individuals. The purpose of this study is to develop a new diagnostic platform using a deep convolutional neural network (DCNN) that can predict the risk of scoliosis progression in patients with AIS.

**Methods:**

Fifty-eight patients with AIS (49 females and 9 males; mean age: 12.5 ± 1.4 years) and a Cobb angle between 10 and 25 degrees (mean angle: 18.7 ± 4.5) were divided into two groups: those whose Cobb angle increased by more than 10 degrees within two years (progression group, 28 patients) and those whose Cobb angle changed by less than 5 degrees (non-progression group, 30 patients). The X-ray images of three regions of interest (ROIs) (lung [ROI1], abdomen [ROI2], and total spine [ROI3]), were used as the source data for learning and prediction. Five spine surgeons also predicted the progression of scoliosis by reading the X-rays in a blinded manner.

**Results:**

The prediction performance of the DCNN for AIS curve progression showed an accuracy of 69% and an area under the receiver-operating characteristic curve of 0.70 using ROI3 images, whereas the diagnostic performance of the spine surgeons showed inferior at 47%. Transfer learning with a pretrained DCNN contributed to improved prediction accuracy.

**Conclusion:**

Our developed method to predict the risk of scoliosis progression in AIS by using a DCNN could be a valuable tool in decision-making for therapeutic interventions for AIS.

## Background

Adolescent idiopathic scoliosis (AIS) is a three-dimensional spinal deformity that predominantly occurs in girls aged between 10 and 13 years that is diagnosed by a coronal plane angle (measured by the Cobb method) of more than 10 degrees [1], without congenital, neuromuscular, or symptomatic etiology. Approximately 2–4% of children under the age of 16 years develop scoliosis, among which only 0.3–0.5% have a progressive curve that requires treatment [2–4]. Severe scoliosis is associated with low back pain, functional disability, cardiopulmonary disorders [5], early degenerative changes in the spine, and mental impairment [6]. In general, the risk of curve progression is most likely to occur during the prepubertal period of peak height velocity (PHV). Therefore, skeletally immature patients with AIS should be carefully monitored using serial examinations and radiographic studies. Conservative treatment with rigid bracing is indicated for patients with residual skeletal growth [2] and a curve size of more than 20–25 degrees [4, 7, 8] until skeletal maturation is achieved to control the progression of scoliosis. Surgical treatment with spinal fusion is generally performed for patients with a curve of more than 40–50 degrees. As the curve progresses, highly invasive spinal fusion surgery is required with the risk of complications. Therefore, early diagnosis and appropriate intervention are important to prevent curve progression.

Regular follow-up with radiography is necessary for patients with relatively mild scoliosis between 10 and 25 degrees. It is widely accepted that skeletal growth and maturation influence the progression of AIS, and the risk of curve progression was found to be significantly correlated with time to PHV [9]. Maturity of the skeleton and PHV can be evaluated by menarche in girls, Tanner staging of sexual maturation, and various radiographic parameters, including the Risser sign [10], Tanner-Whitehouse scores [11], distal radius and ulna stages [12], digital skeletal age scores [9], and Sanders simplified the skeletal maturity system [13]. However, accurate prediction of curve progression remains elusive because the prognosis of the deformity varies in each individual [14, 15]. To prevent future curve progression, determining the risk of scoliosis prognosis and the potential benefit of treatment with orthosis or brace is the responsibility of the primary clinician [16]. Therefore, it may be clinically valuable to accurately predict the risk of scoliosis progression as early as possible.

Rapidly developing machine-learning technology can be applied to automatically determine the risk of scoliosis progression. Artificial neural networks are a set of techniques and algorithms for discovering complex patterns in large datasets, known as deep learning [17]. These models are being widely applied to form state-of-the-art approaches in various areas, such as speech recognition [18], visual object recognition [19], neuronal cell biology [20], and genomics [21, 22]. In particular, the deep convolutional neural network (DCNN) [23], a type of hierarchical neural network, has emerged as a powerful solution that excels in learning the features of objects from large amounts of training images and has successfully improved decision making in several clinical settings, such as diagnosis in radiation oncology [24] and diabetic retinopathy [25], detecting gastric cancer with endoscopic images [26], and the prediction of prognosis [27]. A large number of training images are essential for the successful development of the DCNN. However, owing to the limited number of patients, especially for medical images of rare diseases, it is difficult to collect sufficient datasets to train the network. Transfer learning, which utilizes pretrained models, is adequate for solving this problem. Pre-training with a vast majority of natural images, such as ImageNet [28], can improve the learning accuracy of the DCNN and accelerate its processing.

In this study, we sought to build a diagnostic platform to predict scoliosis curve progression in patients with AIS using a pre-trained DCNN. To achieve this goal, X-rays of patients with AIS were used as training images to learn the progression or non-progression of scoliosis. Our deep learning model achieved a diagnostic accuracy superior to that of spine surgeons. This diagnostic platform will be beneficial for specialists and non-expert clinicians in decision-making for therapeutic interventions for scoliosis.

## Methods

**Fig. 1:**
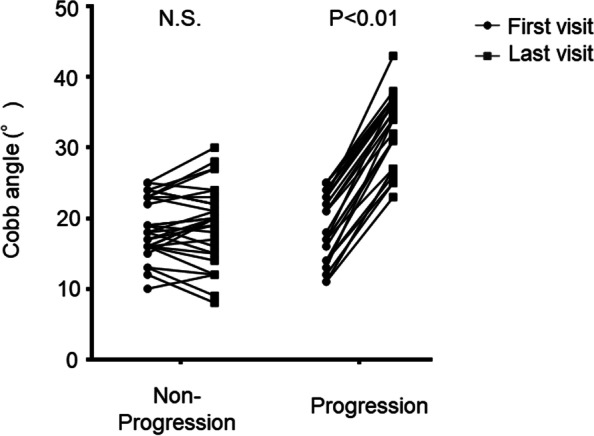
Change in Cobb angle between the initial examination and final follow-up

**Fig. 2:**
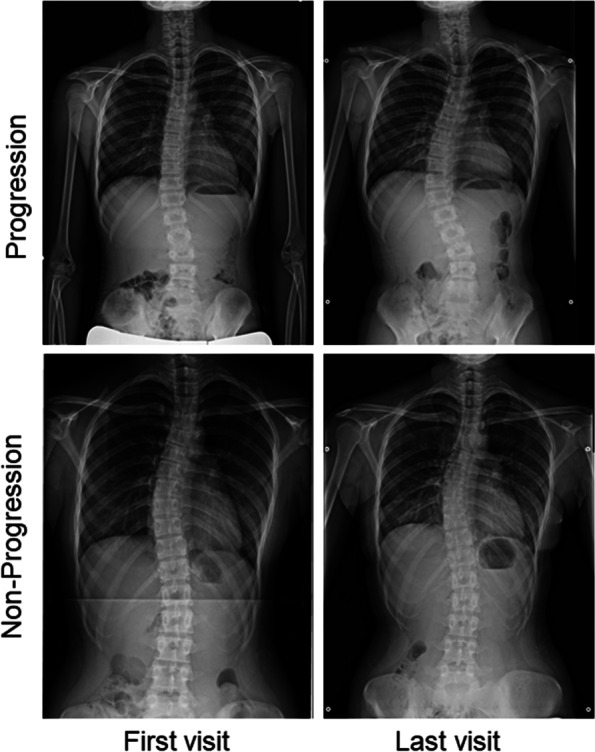
Total spine radiographs in representative cases from the progression and non- progression groups

This study was approved by the ethics committee of Toyama University Hospital. We retrospectively reviewed the data of patients who visited or were referred to our university hospital for AIS screening between 2012 and 2021. All patients received total spine radiograms and standing frontal and sagittal views were used for analysis. The study included a total of fifty-eight patients (49 females and 9 males; mean age: 12.5 ± 1.4 years) with a Cobb angle between 10 and 25 degrees (mean angle: 18.7 ± 4.5). The patients were divided into two groups: those whose Cobb angle increased more than 10 degrees within two years (progression group: 28 patients, 23 females and 5 males; mean age: 12.2 ± 1.6 years) and those whose Cobb angle changed by less than 5 degrees within two years (non-progression group: 30 patients, 26 females and 4 males; mean age: 12.8 ± 1.0 years) (Figs. 1 and 2, Table 1). Because most of the patients who enrolled in this study had non-constructive scoliotic curvature, the Cobb angle could vary with posture and examination time. Therefore, patients with Cobb angle changes between 5 and 10 degrees were excluded from the study to clarify the predictive criteria. Bracing therapy was initiated if a patient developed a Cobb angle of greater than 25 degrees and a Risser sign of less than two or if the curve progressed more than 5 degrees at the semiannual follow-up. Contrarily, bracing therapy was terminated for patients with a Risser sign of 3 or greater and no worsening of the Cobb angle at the semiannual follow-up. The average follow-up duration in the non-progression group was 3.9 ± 1.2 years, and that in the progression group was 2.0 ± 0.9 years.

**Table 1:** Demographics

	Overall	Non-Progression	Progression	*p*-value
	(*n* = 58)	(*n *= 30)	(*n* = 28)	
Age	12.5 ± 1.4	12.8 ± 1.0	12.2 ± 1.6	0.09
Follow up time (year)	3.0 ± 1.4	3.9 ± 1.2	2.0 ± 0.9	< 0.01
Sex (M/F)	9/49	4/26	5/23	N.A
Cobb angle (degree)				
First visit	18.7 ± 4.5	18.6 ± 4.1	18.8 ± 4.9	0.87
Last visit	25.7 ± 8.6	19.1 ± 5.4	32.8 ± 4.9	< 0.01
Increament	6.8 ± 7.1	0.5 ± 2.8	13.6 ± 2.5	< 0.01
Lumbar lordosis (degree)				
First visit	43.8 ± 11.0	41.4 ± 12.3	46.4 ± 10.8	0.10
Thoracic kyphosis (degree)				
First visit	20.7 ± 9.4	20.3 ± 9.4	21.2 ± 9.5	0.75
Risser sign				
Grade 0	12 (20.7%)	4 (13.3%)	8 (28.6%)	0.49
Grade 1	15 (25.9%)	9 (30%)	6 (21.4%)	
Grade 2	8 (13.8%)	6 (20%)	2 (7.14%)	
Grade 3	9 (15.5%)	5 (16.7%)	4 (14.3%)	
Grade 4	11(19%)	5 (16.7%)	6 (21.4%)	
Grade 5	3 (5.1%)	1 (3.3%)	2(7.14%)	
Pre-menarche patients	20 (34.4%)	6 (20.0%)	14 (60.8%)	0.002
Month scince menarche	21.5 ± 13.3	21.7 ± 14.2	21.0 ± 11.4	0.89

*M* Male, *F* Female

**Fig. 3:**
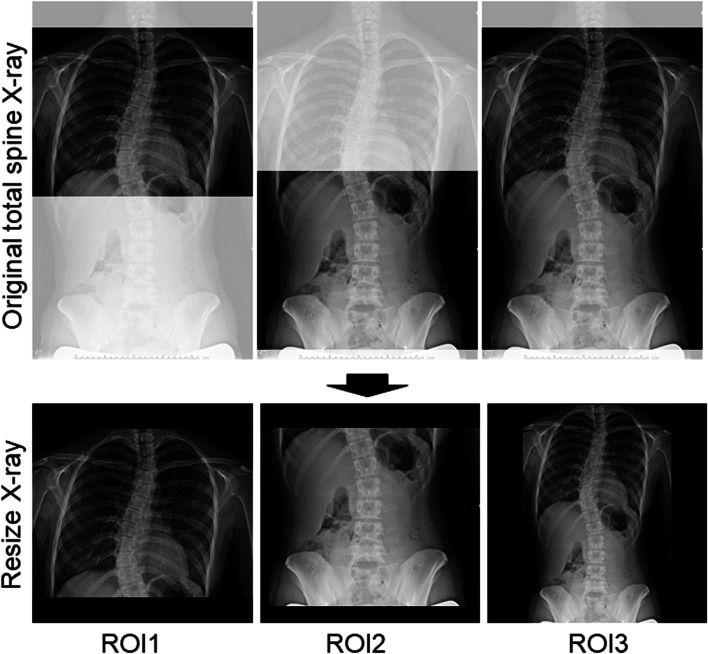
Frontal view of total spine radiographs for prediction of curve progression by DCNN. Images were cropped into three different regions of interest (ROI); C7 vertebra to diaphragm (ROI1, lung), diaphragm to iliac (ROI2, abdomen), and C7 vertebra to iliac (ROI3, total spine). DCNN, deep convolutional neural network

The frontal view of the total spine radiographs was used as the source data for learning and prediction by the DCNN. Images were cropped using a combination of three horizontal lines and two vertical lines passing through the acromion on both sides, and images using three regions of interest (ROI) were prepared: the C7 vertebra and diaphragm (ROI1, lung); diaphragm and ilium (ROI2, abdomen); and C7 vertebra and ilium (ROI3, total spine) (Fig. 3). Each cropped image was resized while maintaining the aspect ratio such that the long side was 299 pixels according to the input size of the pre-trained DCNN (Xception) with ImageNet [29]. Black pixels were inserted on the short side to obtain a resized image with 299 × 299 pixels. Computational processing was performed using a CPU: Intel Corei7—10,700, memory: 128 GB, GPU: NVIDIA Quadro RTX 8000, and MATLAB 2020a. In the learning by Xception, fine-tuning was conducted using the weights by pre-training on ImageNet, and leave-one-out cross-validation was used for evaluation. In some experiments, the accuracy of scoliosis progression prediction was assessed with or without transfer learning to evaluate the effect of the pre-trained DCNN and fine-tuning. The training conditions were as follows: learning rate, 0.001; batch size, 8; number of epochs, 50; and optimizer, stochastic gradient descent with momentum (SGDM). The gradient-weighted class activation mapping (Grad-CAM) method was used to visualize the regions recognized by the DCNN [30]. First, ROI3 was divided into six regions: the upper to middle thoracic spine, lower thoracic spine, lumbar spine, lung, abdomen, iliac, and others. Next, the regions in which the heat map gave the strongest signal were compared between the successfully and unsuccessfully classified groups (Fig. 6). Five spine surgeons also predicted the progression of scoliosis by reading the X-rays in a blinded manner. Frontal radiographs of ROI3 images from all 58 patients with AIS were used for the prediction and personal information such as patient gender, age, or presence or absence of menarche was not provided.

Data are presented as mean and standard deviation. Significant differences between means were analyzed using the t-test (two-sided) and the chi-square test. In comparing AUC (area under the curve) among the three types of ROI, we used analysis of variance (ANOVA) followed by Tukey–Kramer post hoc test. In addition, the Pearson correlation test was used to analyze the correlation between the curve progression and several parameters. Statistical analysis was performed using Excel statistical software (Statcel4; OMS, Tokorozawa, Japan) and GraphPad Prism (GraphPad Software, San Diego, CA, USA). Classification accuracy was evaluated using receiver operating characteristic (ROC) analysis. A computer program (LABMRMC, The University of Chicago, Chicago, IL) was used to obtain ROC curves and evaluate statistically significant differences [31, 32]. Statistical significance was set at *P *< 0.05.

## Result

**Fig. 4:**
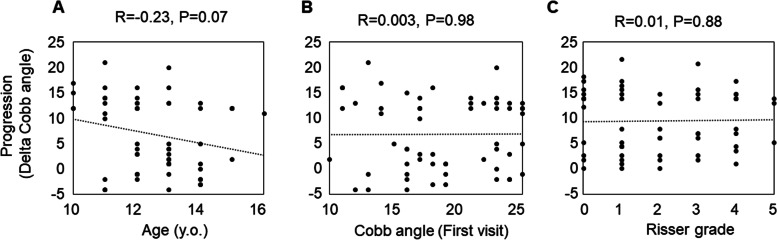
The correlation coefficient between the patient profiles at the first visit and the curve progression. **A**, age; **B**, Cobb angle at first visit; **C**, Risser grade. The Pearson correlation test was used

In the progression group, the Cobb angle increased by an average of 13.6 degrees within two years, whereas in the non-progression group, the Cobb angle only increased by 0.5 degrees about four years (Table 1). The two groups demonstrated no significant difference in sagittal radiographic alignment, such as lumbar lordosis and thoracic kyphosis. In contrast, the percentage of pre-menarche patients was significantly higher in the progression group than in the non-progression group (Table 1). This cohort included 76% skeletally immature patients with Risser signs of 3 or less, and 34% of the females were premenarcheal. The correlation between the patient profiles at the first visit and curve progression was also evaluated. There was a weak negative correlation between scoliosis progression and age (*r* = -0.23, *p* = 0.07) at the first examination; however, there was no correlation between scoliosis progression and Cobb angle (*r* = 0.003, *p* = 0.98), lumbar lordosis (*r* = 0.199, *p* = 0.13), thoracic kyphosis (*r* = -0.01, *p* = 0.91), or Risser sign (*r* = 0.01, *p* = 0.88) (Fig. 4, Table 2).

**Table 2:** Correlation between Cobb angle and each parameter

	Cobb angle progression	
	Correlation	*p*-value
Age	-0.230	0.07
Cobb angle	0.003	0.98
Lumbar lordosis	0.199	0.13
Thoracic kyphosis	-0.010	0.91
Risser sign	0.010	0.88

**Fig. 5:**
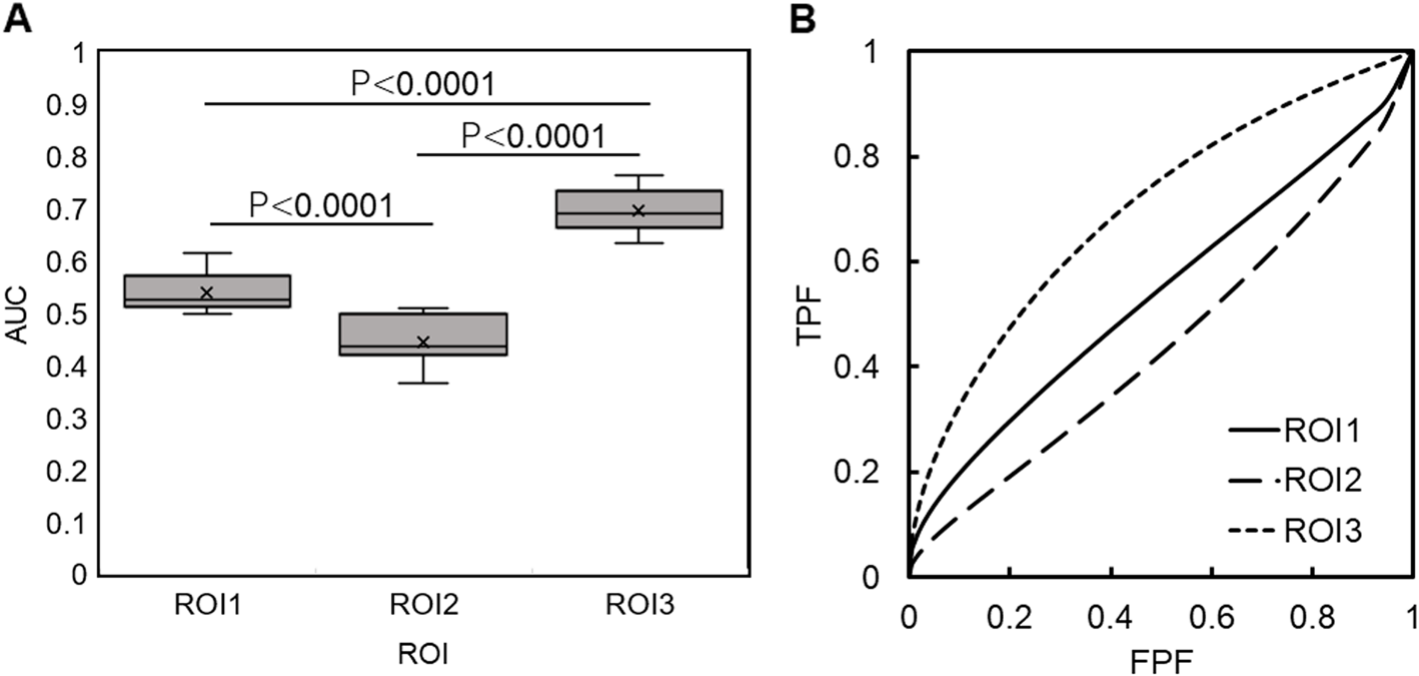
Comparison of AUC for three different ROIs (**A**) and ROC curves (**B**). Error bars denote the means ± SD. Statistical analysis was performed using one-way ANOVA. AUC, area under the curve; ROI, region of interest; ROC, receiver operating characteristic; SD, standard deviation ; TPF, true positive fraction; FPF, false positive fraction

**Fig. 6:**
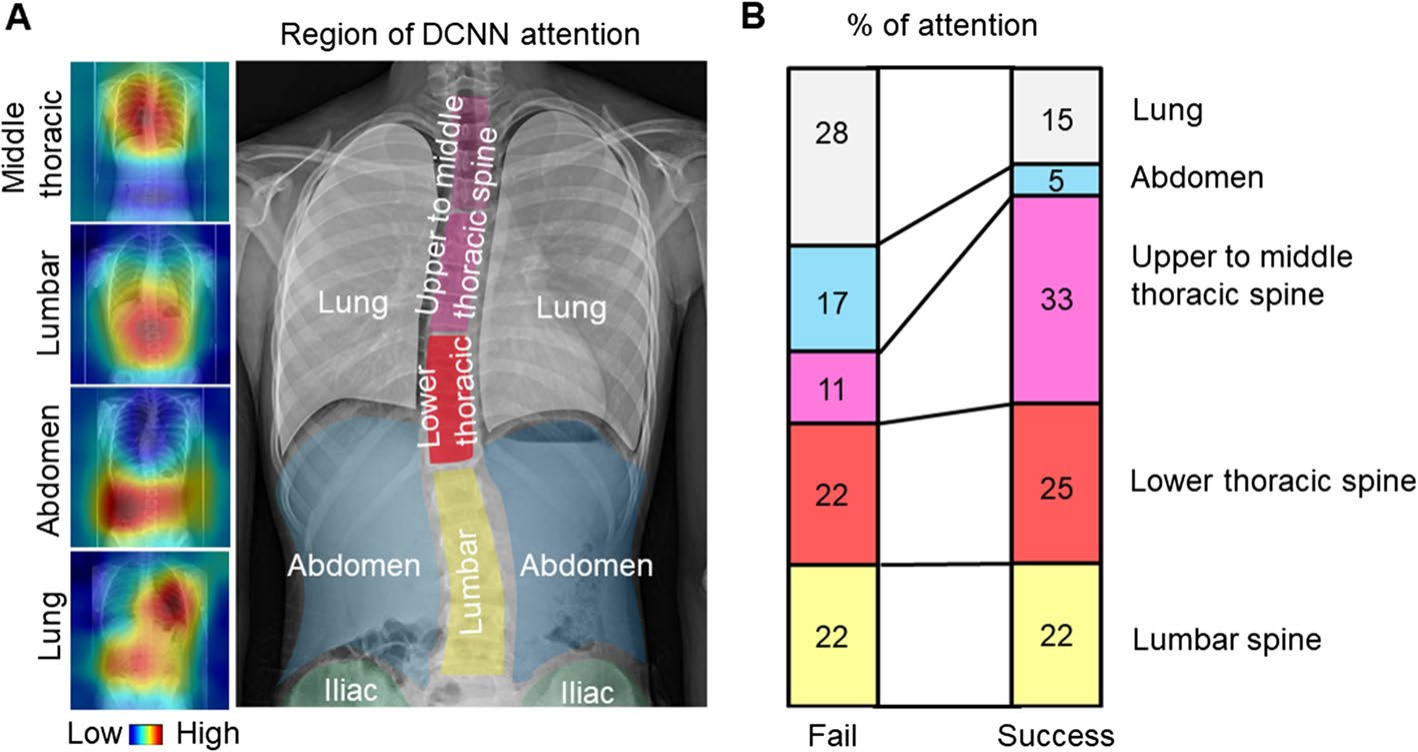
Grad-cam visualization and the region recognized by the DCNN (**A**). Comparison of the regions that DCNN paid the most attention to between successfully and unsuccessfully classified groups (**B**). DCNN, deep convolutional neural network

The performance of the DCNN for the curve progression of AIS using ROI3 achieved a 61% sensitivity, 77% specificity, and 69% accuracy compared to ROI1 (53% sensitivity, 57% specificity, and 55% accuracy) and ROI2 (50% sensitivity, 50% specificity, and 50% accuracy) (Table 3). ROI3 also showed significantly higher AUC (0.7 ± 0.03) than that of ROI1 (0.53 ± 0.04) and ROI2 (0.44 ± 0.04) (Fig. 5). In addition, the results of prediction without transfer learning and fine-tuning showed inferior prediction results for ROI3 (57% sensitivity, 70% specificity, and 64% accuracy) compared to the analysis with pre-trained DCNN and fine-tuning (Table 3). The Grad-CAM heatmap showed the regions that the DCNN paid the most attention to in predicting scoliosis progression using ROI3. In the successfully classified group, Grad-CAM predicted scoliosis progression by focusing on the spinal column in 80% of the cases (33% upper-middle thoracic, 25% lower thoracic, and 22% lumbar). In contrast, non-spinal regions, such as the chest and abdomen, were recognized in the unsuccessful group in 45% of the cases (Fig. 6). Finally, the average accuracy of the five surgeons (47%) was inferior to that predicted by the DCNN (69%) (Table 4).

**Table 3:** Predictive values of curve progression with or without pre-trained deep convolutional neural network and fine-tuning

	AUC	Accuracy	Specificity	Sensitivity
Pre-trained DCNN				
ROI1	0.53	0.55	0.57	0.53
ROI2	0.44	0.5	0.5	0.5
ROI3	0.7	0.69	0.77	0.61
Without transfer learning				
ROI3	0.68	0.64	0.7	0.57

*ROI* Reagion of interest, *AUC* Area under the curve

**Table 4:** Predictive values of curve progression by orthopedic surgeons

	Accuracy	Specificity	Sensitivity
Surgeon 1	0.50	0.52	0.46
Surgeon 2	0.54	0.65	0.43
Surgeon 3	0.51	0.54	0.48
Surgeon 4	0.42	0.48	0.30
Surgeon 5	0.40	0.44	0.36
Average	0.47 ± 0.06	0.52 ± 0.08	0.40 ± 0.07

## Discussion

The deep learning system developed in this study could predict the progression of scoliosis with the highest accuracy of 69% and an AUC of 0.7. In addition, the DCNN could predict the progression of scoliosis with higher accuracy than that of spine surgeons. Transfer learning with a pretrained DCNN contributed to improved prediction accuracy.

Over the past decades, various indicators, including demographic, radiographic, physiological, biochemical, and their combinations, have been reported to be significantly associated with the progression of spinal deformity in AIS. Patients younger than 12–13 years old at the time of diagnosis have a significantly higher risk of developing spinal deformities [33, 34]. The risk of progression increases with a curve of more than 25 degrees [35], the flexibility of less than 28% [36], and patients with an immature skeleton (based on radiographic criteria) had a significantly higher risk of curve progression [8]. In addition, the progression of spinal deformity is also related to the wedging of the intervertebral discs and adjacent vertebral bodies, as well as longitudinal overgrowth of the vertebral bodies due to disproportionate endochondral-membranous bone growth [37–39]. However, the progression of scoliosis does not always coincide with skeletal growth. It is challenging for specialists as well as clinicians to adequately predict which type of scoliosis will progress and require therapeutic intervention, especially in mild scoliosis with a Cobb angle of less than 25 degrees. In fact, the ability of spine surgeon predict prognosis based solely on total spine radiographs at the initial visit was less than 50% in this study.

Skeletally immature patients with mild scoliosis of less than 25 degrees should undergo regular X-ray examinations to monitor the progression of scoliosis [40]. Orthosis or bracing therapy is indicated if the curve progresses more than 5 degrees during follow-up or reaches more than 20–25 degrees [8]. In general, early intervention in mild scoliosis is considered overtreatment because of the chaotic nature of the scoliosis progression [8, 41]. Because scoliosis may progress, stabilize, or disappear, observation is the primary treatment option for skeletally immature patients with mild scoliosis. However, this passive approach may result in missed treatment opportunities in patients with progressive scoliosis and does not address the patient and parents’ anxiety or stress about the progression of the curvature [41, 42]. Therefore, predicting the curve trajectory of a patient with early scoliosis has significant clinical implications. Deep-learning-based prognostic methods can help develop a preemptive treatment strategy that allows for more intensive treatment, such as early bracing or prolonged daily wearing, and early surgical intervention in cases with a high risk of progression, rather than conventional observation.

In this study, the DCNN predicted curve progression with the highest accuracy and AUC using ROI3. Grad-CAM showed that 25% of the successfully classified cases recognized the lower thoracic spine in ROI3. Because the lower thoracic spine is the transition point between the thoracic and lumbar spine, information missing from the chest to the abdomen in ROI1 and ROI2 may have contributed to the low prediction results. Grad-CAM computes gradients by back-propagating the prediction scores to the target layer of the network, and combines the forward feature maps by fitting them as weights. However, these weights do not correctly evaluate the importance of the feature map, and the feature map itself contains noise and includes unimportant parts [43]. Therefore, building an “explainable” predictive model that can adequately interpret the decisions made by the DCNN is critical to improving accuracy.

Only a few studies have investigated the use of deep learning to predict scoliosis progression. Wang et al. built a very accurate scoliosis prediction model using CapsNet [44]. However, their study limited the input images to only the major thoracic curves and did not examine the effect of double or triple major curves on the risk of progression. Although the prediction accuracy of the present study is inferior to that of their study, the present study targeted the entire spine and included more imaging information, such as trunk and rib rotation, pelvic tilt, and spinal deformity. Previous reports have shown that apical vertebral rotation and rib vertebra angle are possible parameters to predict the prognosis of trunk imbalance [45, 46]. Therefore, DCNN, which excels in image recognition, would favor full spine X-rays that include the thorax, ribs, clavicles, and spine in predicting progression. However, the sensitivity, specificity, and accuracy of this pilot study have not reached sufficient levels for clinical application. As we increase the number of cases, we hope to improve the accuracy of our model in identifying the characteristics of scoliosis progression, which will allow us to build a more accurate prediction model based on simple X-rays during the early stages of scoliosis. In addition, our model does not incorporate additional clinical parameters, such as age, sex, growth rate, or measures of skeletal maturity (Risser scoring, Saunders stage, and distal radius and ulna stage). The prediction accuracy can be improved by inputting X-ray images and patient profiles during the initial examination. Further clinical validation will facilitate the application of predictive models to support objective clinical decisions using the DCNN.

When developing deep learning frameworks for medical images, both the volume and quality of the data must be considered. Particularly in the case of rare diseases, it may be impossible to collect enough patients or samples. Transfer learning and fine-tuning can be adapted even when the dataset is small. Transfer learning is a powerful and promising technique that uses pretrained DCNN models to handle tasks with different patterns. For example, transfer learning has been successfully used for brain tumor recognition [47], pneumonia diagnosis [48], and seizure classification [49]. Pre-trained DCNN uses the parameters of the network on which DCNN was trained as the initial values. On the other hand, fine-tuning can adjust the parameters of a portion or all the layers of a trained model. Pre-training and fine-tuning are known to provide better results. In this study, the combination of both methods predicted scoliosis progression better than analysis without pre-training and fine-tuning.

The present study had several limitations that should be addressed in future studies. We only considered whole spine X-rays of 58 patients with AIS in this pilot study; integrating and analyzing the results in a larger study population would allow us to develop a more accurate system. Furthermore, although only Japanese patients with AIS were analyzed in this study, it is essential to establish the feasibility of this study among different racial groups. Furthermore, the accuracy of this prediction model must be verified through prospective follow-up studies. Finally, the ensemble method integrates multiple learning algorithms and obtains better prediction performance than using a single learning algorithm.

## Conclusions

Successful treatment of AIS remains a challenging and complex issue for orthopedic surgeons. Therefore, we developed a clinically accessible predictive model for the risk of disease progression that can facilitate the planning of timely interventions to treat AIS during early clinical visits. In addition, predicting the prognosis of skeletally immature patients with mild scoliosis can help establish new evidence for early therapeutic intervention.

## Data Availability

The datasets analyzed in this study are not available to the public for privacy reasons. However, the data supporting the findings of this study are available from the corresponding author on reasonable request.

## Abbreviations

AIS: Adolescent Idiopathic Scoliosis
DCNN: Deep Convolutional Neural Network
ROI: Regions Of Interest
PHV: Peak Height Velocity
Grad-CAM: Gradient-weighted Class Activation Mapping
SGDM: Stochastic Gradient Descent with Momentum
AUC: Area Under the Curve
ANOVA: Analysis Of Variance
ROC: Receiver Operating Characteristic
